# Anesthetic Evaluation and Perioperative Management in a Patient with New Onset Mediastinal Mass Syndrome Presenting for Emergency Surgery

**DOI:** 10.1155/2011/782391

**Published:** 2011-12-22

**Authors:** David Shi, Christopher A. J. Webb, Meredith Wagner, Anis Dizdarevic

**Affiliations:** ^1^Department of Anesthesiology, Columbia University College of Physicians & Surgeons/Columbia University Medical Center, New York, NY 10032, USA; ^2^Department of Anesthesiology, Columbia University College of Physicians & Surgeons/Columbia University Medical Center, Presbyterian Hospital Room 505, 622 West 168th Street, New York, NY 10032, USA

## Abstract

Mediastinal mass syndrome (MMS) is a complex case that poses many challenges to the anesthesiologist. The cornerstone of management focuses on the potential hemodynamic changes associated with this syndrome. We describe the anesthetic management of a patient with a previously undiagnosed mediastinal mass presenting for emergency neurosurgical surgery.

## 1. Introduction

Mediastinal mass syndrome (MMS), as initially described by Bittar and colleagues in the 1970s, is associated with significant risk for perioperative morbidity and mortality that continues to challenge the anesthesiologist [1, 2]. Patients who present with MMS are of a particular concern due to the varying degrees of clinical presentation ranging from patients who are completely asymptomatic to those who present with complete cardiovascular or cardiopulmonary collapse. Unlike pediatric patients, the adult patient with MMS is associated with less perioperative complications [3, 4]. Nonetheless, adults with MMS presenting for emergency surgery heighten the anesthetic risk. We present the unique case of a 23-year-old female who presented to the emergency room with MMS in the context of spinal cord compression.

## 2. Case Description

A 23-year-old, previously healthy woman presented to the emergency room with new onset bilateral lower extremity weakness along with several months of worsening chest pain, left upper quadrant abdominal pain, and intermittent back pain. Computerized axial tomography (CAT) of the chest with intravenous contrast showed a 4.9 × 7.6 × 5.8 cm anterior mediastinal mass, a 2.6 × 3.7 cm mass above the right pulmonary artery, a 1.3 × 3.7 cm mass just below the carina, and a 1.5 × 2.6 cm mass along the left prevascular space along the aortic arch. Compression of the proximal or distal airways was not noted. MRI confirmed vertebral involvement and extension of the soft tissue mass into the epidural space with corresponding spinal cord compression. Neurosurgery and Anesthesiology were consulted for emergency surgical decompression of the thoracic spine.

On exam, the patient was in a semisupine position with the head of bed elevated to greater than 30 degrees. Although the patient was highly anxious with labored breathing, she was in minimal distress. No facial edema, cyanosis, wheezing, or stridor was noted. She endorsed mid-sternal chest pain that was nonpositional and nonpleuritic. Placement in the right and left lateral decubitus positions neither worsened nor improved her symptoms and vitals remained stable. While placement in the supine position worsened her back pain, her chest pain and breathing remained unchanged. Given her hemodynamic stability along with the above signs and symptoms, her MMS was determined to be mild-moderate. Imaging did not show any tracheobronchial compression or any obvious involvement of the vena cava, suggesting that her anesthetic risk in the context of MMS was determined to be uncertain. To complicate matters, her neurological exam continued to worsen with the inability to raise her legs against gravity, and she had just eaten a full meal within an hour before presenting to the emergency room. 

Prior to induction, cardiothoracic surgery was notified and a rigid bronchoscope was available in the operating suite. The patient was premedicated with 10 milligrams of intravenous metoclopramide and 30 mL of sodium citrate orally. Upon arrival to the operating suite, Standard American Society of Anesthesiology (ASA) monitors were applied along with a preinduction radial arterial line and two large bore peripheral intravenous lines. 

The patient was preoxygenated with the head of bed elevated and spontaneous ventilation maintained with midazolam 2 mg, ketamine 70 mg, etomidate 6 mg, and boluses of remifentanil totaling 60 mcg. Prior to direct laryngoscopy, the resident anesthesiologist experienced rigidity upon opening the mouth, which resolved with propofol 50 mg with 20 mg of succinylcholine. Cricoid pressure was continuously applied. The patient was easily intubated with a size 8.0 endotracheal tube with direct visualization. Patient was hemodynamically stable throughout the induction and intubation process. Less than a minute after the initial dose of succinylcholine, a subsequent dose of 50 mg of succinylcholine was given to assess for possible hemodynamic changes in response to paralysis. Shortly after administering succinylcholine, the patient experienced a sudden drop of heart rate to the 20s with gradual decline of systolic blood pressure to the 70s. Atropine 0.4 mg was given with increase in heart rate and return of systolic blood pressure to the 120s. The decision was made to avoid paralysis in the advent that airway or vascular compromise developed and required rapid awakening from anesthesia. The patient was initially ventilated with low tidal volumes to ensure that positive pressure ventilation did not precipitate hemodynamic compromise; the patient was then placed on volume controlled ventilation with low tidal volumes of 6-7 mL/kg to minimize reductions in preload. Peak airway pressures remained less than 25 cm H_2_O. The patient was placed in the prone position without any changes in hemodynamics. She was maintained on remifentanil 2 mcg/kg/min, desflurane, nitrous oxide, and 40% oxygen. The remainder of the case was uneventful and the surgery lasted for 2 hours and 30 minutes with general anesthesia time being 3 hours. The patient remained hymodynamically stable throughout the surgical procedure and received a total of 2 liters of lactated ringers with a total blood loss of 100 mililiters. At the end of the procedure, the patient was placed in the supine position and extubated in the operating room. The patient recovered in the neurosurgical intensive care unit and was transferred to the hematology/oncology floor on postoperative day 3, with minimal paresthesias and improving lower extremity weakness. She was ultimately diagnosed with diffuse B-cell lymphoma and initiated on cyclophosphamide, doxorubicin, vincristine, prednisolone, and rituximab (CHOP-R) chemotherapy during her hospitalization.

## 3. Discussion

Bittar et al. were the first to describe the anesthetic risks and complications during general anesthesia in patients with mediastinal masses [1]. Since that time, many case reports and literature reviews have elucidated the most appropriate preoperative evaluation and/or tests as well as the best treatment modalities for surgical patients who present with mediastinal mass syndrome (MMS). To our knowledge, our case report is the first to describe the anesthetic management of a patient with a previously undiagnosed mediastinal mass presenting for emergency neurosurgical surgery. 

Spinal cord compression in the context of deteriorating neurological function represents a true neurosurgical emergency, limiting the amount of time for a thorough preoperative evaluation. Clinical examination often provides the first diagnostic sign of MMS including cough, dyspnea, hoarseness, difficulty speaking, or syncope with positional changes. Superior vena cava (SVC) syndrome, which includes signs and symptoms of edema of the upper body, dyspnea, headache, changes in vision, reddish face, or cheeks, is an indicator of the mediastinal mass compressing against the SVC [2]. 

However, a lack of symptoms during clinical examination does not guarantee an uneventful anesthetic course once general anesthesia is initiated [5, 6]. Therefore, other modalities, such as chest X-ray, computed tomography (CT), spirometry, flow volume loops, transthoracic echocardiography, and transesophageal echocardiography (TEE), have been proposed and studied for the evaluation of anterior mediastinal masses. During the evaluation, it is important to identify the location of the mass, its relationship and effects to other surrounding structures, the extent of tracheal and vascular compression, patient's cardiopulmonary status, and patency of the airway at both the tracheal and bronchial level. In our patient, preoperative imaging demonstrated encroachment but not compression of the cardiopulmonary structures. Previous case reports have described the use of intraoperative TEE to aid in both surgical planning as well as anesthetic management [7, 8]. Given that our patient only demonstrated encroachment of the cardiopulmonary vasculature and she was going to be placed in the prone position with a prone view, the decision was made to not utilize intraoperative TEE. 

While spirometry and flow volume loops have been classically included in the preoperative evaluation, clinically, these studies have shown minimal correlation with the extent of the disease and have not provided valuable information for patient management [9, 10]. However, studies have shown that CT scans demonstrate usefulness in managing the patient; when tracheal compression is greater than 50%, complications with general anesthesia can be expected, especially in children [11, 12]. In an emergent situation, such as with this patient, clinical examination, basic laboratory tests, electrocardiogram, and computed tomography scan are often sufficient for preoperative evaluation and can be obtained in a minimal amount of time.

For patients who require general anesthesia, continuous monitoring of gas exchange and hemodynamics during the induction process is critical. Maintenance of spontaneous ventilation and avoidance of muscle relaxant until the airway is secured and adequate gas exchange can be established with positive pressure ventilation is recommended [12]. Anesthesia induction can be accomplished with either inhalation agents or intravenous titration of ketamine with or without other intravenous agents to maintain spontaneous ventilation. Awake fiberoptic intubation is also a recommended option but rate of success will depend on the techniques and experience of the anesthesiologist. In an emergent and full-stomach scenario, such as this patient, premedications with sodium citrate and metoclopramide along with cricoid pressure can minimize morbidity due to aspiration. 

Despite maintenance of spontaneous ventilation, a case report of airway collapse has been reported [6]. Therefore, it is critical to be prepared to reposition the patient and have additional equipment, such as rigid bronchoscope and extracorporeal membrane oxygenator, available [6, 13]. The utilization of cardiopulmonary bypass as a “stand-by” during induction of anesthesia has been controversial. While neurological injury may not be preventable due to the amount of time needed to establish cardiopulmonary bypass after a respiratory or cardiovascular collapse has occurred, an experienced team may prevent further morbidity or mortality. 

Once the airway is established, a trial of controlled ventilation should precede the usage of muscle relaxant if paralysis is necessary for the procedure. For this patient, 50 mg dose of succinylcholine resulted in cardiovascular compromise, presumably due to the Bezold-Jarisch reflex, and further muscle relaxant was avoided. The patient was maintained on an intravenous/inhalation anesthesia technique with stable hemodynamics throughout the case. 

 Another possible cause of the bradycardia could be a direct stimulation of the parasympathetic system by succinylcholine or its metabolites on cardiac muscarinic receptors [14]. The initial studies of succinylcholine-induced bradycardia suggest that both the timing and the amount of subsequent doses of succinylcholine result in parasympathetic stimulation of the cardiovascular system [15, 16]. In regards to timing, Williams et al. demonstrated that a second dose of succinylcholine given more than 2 minutes after the initial dose results in bradycardia while doses given less than 2 minutes were less likely to cause a profound vagotonic effect [15]. Similarly, Lupprian and Churchill-Davidson illustrated that the parasympathetic response associated with succinylcholine is the dependent on subsequent doses being larger than 50 mg rather than the amount of the initial dose or the number of subsequent doses [16]. Based on the aforementioned studies, it would be surprising but not unlikely that the cause for the bradycardia exhibited in our patient was simply due to the effect of succinylcholine acting as an agonist on the cardiac muscarinic receptors.

In summary, we have described the anesthetic management of a patient with a previously undiagnosed mediastinal mass presenting for emergency surgery. Mediastinal mass syndrome (MMS) is a complex case that poses many challenges to the anesthesiologist. The cornerstone of management focuses on the potential hemodynamic changes associated with this syndrome. The implications of MMS and the preoperative evaluation are discussed.
